# Ultrasonic-Catalyzed Oxidation and Dissolution of Tin Using Hydrogen Peroxide

**DOI:** 10.3390/molecules30071591

**Published:** 2025-04-02

**Authors:** Dongbin Wang, Tian Wang, Shixing Wang, Hongying Xia, Wenlong Miao, Thiquynhxuan Le, Libo Zhang

**Affiliations:** 1 Faculty of Metallurgical and Energy Engineering, Kunming University of Science and Technology, Kunming 650093, China; wangdbkust@163.com (D.W.); wtkust@163.com (T.W.); wsxkm@126.com (S.W.); hyxia81@163.com (H.X.); 18579082577@163.com (W.M.); 2 Key Laboratory of Unconventional Metallurgy, Kunming University of Science and Technology, Kunming 650093, China

**Keywords:** tin dissolution, room temperature, ultrasound, enhanced oxidation

## Abstract

The traditional alkaline process used to prepare sodium stannate faces the challenges of high temperature, low utilization rate, and large hydrogen peroxide consumption, which is mainly due to the low oxidation dissolution efficiency of tin. Here, a new process by ultrasonic-enhanced oxidation and dissolution efficiency of tin at room temperature was proposed. The effects of temperature, ultrasonic power, sodium hydroxide concentration, hydrogen peroxide dosage, and ultrasonic time on the oxidation dissolution efficiency of tin were systematically investigated. The results show that the process of ultrasonic-enhanced oxidation dissolution of tin is a new method with high efficiency and low cost. At room temperature, the tin dissolution efficiency was as high as 99.3% under ultrasound, which was 28% higher than that of the conventional method under the same conditions. The introduction of ultrasound promoted the generation of strong oxidizing hydroxyl radicals (·OH) from hydrogen peroxide, significantly improved the surface roughness of the tin sheet from 6.875 μm in the conventional treatment to 34.135 μm in the ultrasonic treatment, and destroyed the passivation layer on the surface of the tin sheet, thereby improving the dissolution efficiency of tin. Compared with conventional tin dissolution conditions, ultrasonic-enhanced oxidation could decrease the reaction temperature by 30 °C, reduce the consumption of sodium hydroxide by 33.3%, and save the consumption of hydrogen peroxide by 15% while achieving the same tin dissolution effect. This new technology provides new ideas for the oxidation and dissolution of this valuable metal.

## 1. Introduction

Sodium stannate is an important chemical raw material, widely used in electroplating, textile, anti-corrosion, and organic synthesis catalyst fields. The preparation methods of sodium stannate mainly include de-tinning, alkali fusion, and alkali dissolution methods [1]. The de-tinning method is a method of producing sodium stannate from secondary tin resources such as tinplate and tin-plating slag as a raw material; however, this method has problems such as low purity of sodium stannate products and large differences in raw material components, which make it difficult to achieve stable industrial production. The principle of the alkali fusion method is to react the cassiterite concentrate with sodium hydroxide at high temperatures to form a molten sodium stannate and then obtain the sodium stannate products after cooling, leaching, impurity removal, filtration, and crystallization. However, this method is associated with challenges such as high energy consumption, high costs, a complex process, and a strong corrosive effect on equipment.

Alkaline dissolution is one of the most widely applied methods. In principle, high-purity refined tin is oxidized by an oxidizing agent in a sodium hydroxide solution to dissolve tin and form a sodium stannate solution. The choice of oxidant is a key factor in determining the efficiency of tin dissolution, which directly affects the quality and production efficiency of sodium stannate. In the traditional alkaline dissolution method, sodium nitrate is used as the oxidizing agent, which requires high temperatures to decompose the nitrate ions. This process can lead to the emission of harmful nitrogen oxide gases, high energy consumption, and environmental pollution. Taninouchi et al. [1] studied the effects of oxidizing agents on tin conversion in a sodium hydroxide solution during the preparation of sodium stannate and reported that iodate ion (IO_3_^−^) was one of the most effective oxidizing agents. However, separating and removing residual iodate ions from the sodium stannate product is challenging. In comparison, oxidizing agents that do not introduce impurity ions, such as oxygen and hydrogen peroxide, offer greater advantages. Due to the limited oxidizing capacity of oxygen, an oxygen pressure method under high-pressure and high-temperature conditions is required to obtain satisfactory results. The oxygen pressure method still faces several challenges, including operational safety concerns, high costs, and stringent equipment requirements. Hydrogen peroxide has become the primary oxidizing agent in modern sodium stannate production due to its stronger oxidizing capacity and environmental friendliness. Low reaction temperatures result in slow heat and mass transfer rates in the reaction system, weakening the oxidation effect of hydrogen peroxide on tin sheets and lowering the dissolution efficiency of tin. Therefore, in the preparation process of sodium stannate, oxidization of tin with hydrogen peroxide generally requires temperatures of 75–85 °C and oxidation times of 3.5–4.5 h [2] and sometimes even requires temperatures of up to 100 °C [3] to obtain satisfactory tin dissolution efficiency. However, hydrogen peroxide is unstable above 30 °C and decomposes into water and oxygen. Its decomposition efficiency increases as temperature rises, resulting in low utilization efficiency and high consumption of hydrogen peroxide. Thus, achieving efficient and rapid tin dissolution at low temperatures while minimizing hydrogen peroxide consumption is a critical issue that needs to be addressed.

Ultrasonic waves are mechanical waves with frequencies above 20 kHz and have complex characteristics such as cavitation and mechanical effects, providing a unique environment for reactions that are typically difficult or impossible to achieve under conventional conditions, thus paving the way for new reaction pathways. The combination of ultrasound with hydrogen peroxide oxidation represents an advanced oxidation process. Xin et al. [4] reported that the introduction of ultrasound intensified the oxidizing power of hydrogen peroxide, thereby enhancing the leaching rates of both zinc and germanium. Studies have indicated that the application of ultrasound strengthens the oxidizing effect of hydrogen peroxide on organic pollutants in sludge [5]. They reported that the cavitation effect of ultrasound facilitated the decomposition of hydrogen peroxide into hydroxyl radicals with stronger oxidizing capabilities, thereby enhancing the oxidative performance of hydrogen peroxide. In addition, ultrasound-assisted tin leaching has also been reported. Liu et al. [6] employed an ultrasound-assisted alkaline leaching process to extract tin from tinplate scrap and reported that ultrasonic leaching at 60 °C for 30 min can achieve the same Sn leaching effect as conventional leaching at 80 °C for 60 min, thus reducing the temperature and saving time. Liu et al. [7] investigated the separation of lead and tin in lead–tin alloys through nitric acid leaching and discovered that the introduction of ultrasound could remove the insoluble passivation film. This promoted contact between tin and nitric acid, thereby significantly enhancing the leaching efficiency of tin. Therefore, it is theoretically feasible to use ultrasound to enhance the oxidation performance of hydrogen peroxide and the solid–liquid reaction of tin, thereby improving the oxidation effect of hydrogen peroxide on tin and achieving efficient dissolution of tin.

In this work, the ultrasonic-enhanced oxidation of tin for efficient preparation of sodium stannate in a NaOH/H_2_O_2_ system at room temperature is studied first, using refined tin as the raw material, sodium hydroxide as the alkali, and hydrogen peroxide as the oxidant. The effects of various reaction parameters on the efficiency of tin dissolution are systematically investigated, the optimal process conditions are established, and the mechanism by which ultrasound enhances tin dissolution at room temperature is elucidated. The research results provide a new idea for the preparation of sodium stannate at room temperature, which is conducive to the dissolution of tin without heating, reducing the volatilization of hydrogen peroxide, improving the effectiveness of hydrogen peroxide utilization, and thereby enhancing the oxidation and dissolution efficiency.

## 2. Results and Discussion

### 2.1. Influence of Reaction Factors on the Effectiveness of Tin Dissolution

#### 2.1.1. Effect of Temperature

**Figure 1:**
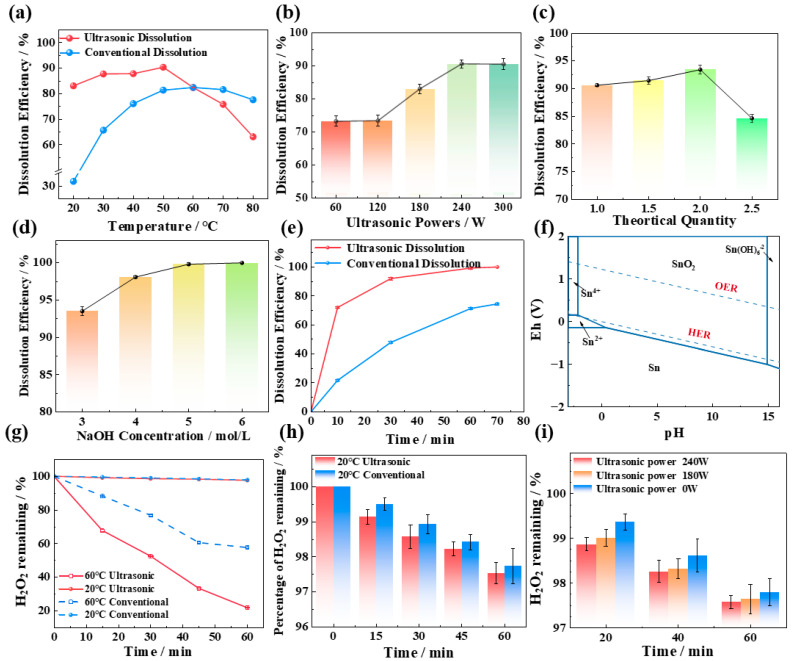
Effects of temperature (**a**), of ultrasonic power (**b**), of H_2_O_2_ addition (**c**), of NaOH concentration (**d**), and of ultrasonic time (**e**) on the tin dissolution efficiency; (**f**) phase equilibrium for Sn in aqueous solution described by the Pourbaix diagram and the oxygen evolution reaction (OER) with the hydrogen evolution reaction (HER); (**g**) the change in the content of H_2_O_2_ in NaOH solution (without tin); (**h**) the change in the content of H_2_O_2_ in NaOH solution at 60 °C (without tin); and (**i**) the change in the content of H_2_O_2_ in NaOH solution at different ultrasonic powers (without tin).

The impact of temperature on tin dissolution was studied under the conditions of 3 mol/L NaOH concentration, 1.5 times the theoretical amount of H_2_O_2_ dosage (as shown in Equation (1)), and a 60 min reaction time. In the ultrasound experiment, the ultrasound power applied was 180 W. The dissolution efficiency of Sn at different temperatures under ultrasonic and conventional conditions is shown in Figure 1a.

Temperature clearly plays a crucial role in the utilization efficiency of H_2_O_2_ and the dissolution effect of tin. At high temperatures (60–80 °C), the effect of the ultrasonic tin dissolution process is not as good as that of the conventional process, as shown in Figure 1a. The reason is that the decomposition of H_2_O_2_ is enhanced at high temperatures, and the decomposition process is more obvious under the action of ultrasound. In the absence of tin, the changes in H_2_O_2_ content in NaOH solutions at various temperatures are illustrated in Figure 1g,h. Under conventional high-temperature conditions (60 °C), after 60 min of treatment, the remaining hydrogen peroxide in the solution is still 63.25% of the initial amount. In contrast, after 60 min of ultrasonic treatment, the remaining H_2_O_2_ is only 21.95% of the initial amount, indicating a significantly higher decomposition efficiency in the presence of ultrasound at the same temperature. This also explains why, as shown in Figure 1a, above 60 °C, the ultrasonic tin dissolution effect is inferior to that of conventional tin dissolution. In addition, other studies have indicated that excessively high temperatures can reduce the number of hydroxyl radicals (·OH) generated by H_2_O_2_ under ultrasonic conditions [8,9]. High temperatures enhance the reaction of pre-existing ·OH with H_2_O_2_ to form hydroperoxyl radicals (O_2_H) (Equation (2)) or promote the dimerization of ·OH to regenerate more stable H_2_O_2_ (Equation (3)). The oxidizing ability of ·O_2_H and H_2_O_2_ is not as good as that of ·OH, which is another reason for the diminished efficacy in tin dissolution.

Sn + 2NaOH + 2H_2_O_2_ → Na_2_SnO_3_·3H_2_O (1)

·OH + H_2_O_2_ → ·O_2_H + H_2_O (2)

2·OH ↔ H_2_O_2_ (3)

Compared with conventional treatments, ultrasonic treatment significantly improves the dissolution of tin at low temperatures (20–50 °C). The reason is that ultrasound promotes the decomposition of hydrogen peroxide in alkaline solutions, leading to the generation of free radicals with strong oxidizing properties, thereby enhancing the oxidizing power of H_2_O_2_. At room temperature (20 °C), the conventional tin dissolution efficiency is 31.85%, whereas the ultrasonic tin dissolution efficiency reaches 83.05%. The tin dissolution efficiency under ultrasound is 51.2% higher than that under conventional conditions and is even higher than the highest conventional dissolution efficiency at 60 °C. The H_2_O_2_ content in NaOH solutions at room temperature in the absence of tin was analyzed. The introduction of ultrasound at room temperature does not promote the loss of hydrogen peroxide due to decomposition, and the H_2_O_2_ content in the solutions is about 98% of the amount added. Therefore, all subsequent experiments in this work were carried out at room temperature.

#### 2.1.2. Effect of Ultrasonic Power

**Figure 2:**
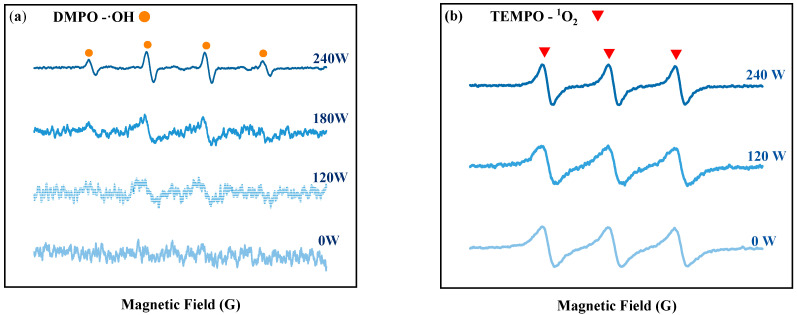
EPR detection results of DMPO-OH (**a**) and of DMPO-^1^O_2_ (**b**).

The impact of ultrasonic power on tin dissolution was studied under the conditions of 3 mol/L NaOH concentration, 1.5 times the theoretical amount of H_2_O_2_ dosage, a 60 min reaction time, and room temperature (20 °C). The dissolution efficiency of Sn at different ultrasonic powers is depicted in Figure 1b. At lower ultrasonic powers (60–120 W), the tin dissolution efficiency exhibited minimal variation. This is because fewer free radicals are produced by hydrogen peroxide in the ultrasonic power range of 60–120 W. The EPR results at various ultrasonic powers can prove this result, as shown in Figure 2a, the signal peaks of ·OH are weak in the range of 60–120 W. When the ultrasonic power increases from 120 to 240 W, the tin dissolution efficiency increases significantly, reaching an optimum value of 90.61% at 240 W. This corresponds to the phenomenon observed in the EPR spectrum in Figure 2a, where the signal peak of hydroxyl radicals (OH) is the strongest at 240 W. Regardless of the presence or absence of ultrasound, the ^1^O_2_ peak exists in the solution, and the peak intensity of its signal peak remains virtually unchanged with variations in ultrasonic power in Figure 2b. This indicates that the change in tin dissolution efficiency is more significantly influenced by ·OH than by ^1^O_2_. The effects of different ultrasonic powers on the hydrogen peroxide content in solution were examined at room temperature. As shown in Figure 1i, when the ultrasonic power is varied within the range of 0–240 W, the residual H_2_O_2_ concentration consistently remains between 97.5% and 97.8% of the initial dosage over a 60 min period. These results clearly demonstrate that the introduction of ultrasound does not lead to significant volatilization loss of H_2_O_2_ at room temperature. Consequently, increasing the ultrasonic power to facilitate the generation of ·OH with strong oxidizing properties is identified as a key factor in improving the dissolution efficiency of tin sheets.

Upon increasing the ultrasonic power to 300 W, the intensifying effect of ultrasound on the tin dissolution efficiency weakens. This is attributed to the fact that excessive ultrasonic power can lead to significant heat generation in the solution, and thermal effects may suppress the formation of ·OH and simultaneously enhance the decomposition of H_2_O_2_. Hu et al. [10] demonstrated that the concentration of hydroxyl radicals increases with increasing ultrasound output power; however, this relationship holds true only within a specific range. When the power exceeds a certain threshold, a reduction in ·OH production may be observed. Consequently, an ultrasonic power of 240 W is selected as the optimal level for this study.

#### 2.1.3. Effect of H_2_O_2_ Addition

The effect of the hydrogen peroxide dosage on the tin dissolution efficiency during the ultrasonic tin dissolution process was examined under the conditions of 3 mol/L NaOH concentration, 60 min reaction time, 240 W ultrasonic power, and room temperature (20 °C). The dissolution efficiency of Sn at different hydrogen peroxide dosages is shown in Figure 1c.

When the stoichiometric ratio of hydrogen peroxide to the theoretical requirement increases from one to two, the tin dissolution efficiency increases from 90.61% to 93.6%. This enhancement is attributed to the probability of contact between tin, alkali, and hydrogen peroxide and the free radicals generated by hydrogen peroxide under ultrasonication increasing, which enhances the oxidative capacity of the reaction system and significantly improves the tin dissolution efficiency [11]. However, when the stoichiometric ratio of hydrogen peroxide further increases to 2.5, a noticeable decrease in the tin dissolution efficiency is observed. Three primary reasons lead to this phenomenon. First, as hydrogen peroxide is continuously added, the oxygen potential in the solution increases. Once the concentration of hydrogen peroxide in the solution reaches the threshold for the oxygen evolution reaction (OER), oxygen begins to evolve, reducing the utilization efficiency of hydrogen peroxide. Wang et al. [12] investigated the decomposition process of hydrogen peroxide and reported that the activation energies for 27.5%, 50%, and 70% hydrogen peroxide were in the ranges of 64.87–119.62 kJ/mol, 50.19–75.19 kJ/mol, and 25.00–74.59 kJ/mol, respectively. The higher the concentration of hydrogen peroxide in the solution is, the lower the activation energy is, which means that the more reactive hydrogen peroxide is, the more violent its decomposition reaction is, and the easier it is to decompose into oxygen. Second, at high concentrations, hydrogen peroxide can act as a scavenger for free radicals, especially for ·OH. An excessively high concentration of H_2_O_2_ is conducive to its recombination with ·OH, thereby reducing the amount of ·OH in the solution and diminishing the oxidative capacity of the reaction system. Consequently, it is not advisable to add hydrogen peroxide in excess [13]. Third, in the ternary synthesis process involving Sn, H_2_O_2_, and NaOH, an excessive amount of H_2_O_2_ directly results in a significant decrease in the concentration of the reactant sodium hydroxide. As depicted in Figure 1f, the lower the alkalinity of the solution is, the more challenging it is for the tin sheets to dissolve and convert into sodium stannate. In conclusion, the optimal ratio of hydrogen peroxide addition to the theoretical value is two.

#### 2.1.4. Effect of NaOH Addition

The impact of NaOH addition on the tin dissolution efficiency during the ultrasonic tin dissolution process was investigated under the following conditions: two times the theoretical amount of H_2_O_2_ dosage, a 60 min reaction time, 240 W ultrasonic power, and room temperature (20 °C). The dissolution efficiency of Sn with different NaOH concentrations is shown in Figure 1d.

The NaOH concentration is a critical factor influencing the dissolution efficiency of tin. The experimental results indicate that the tin dissolution efficiency increases with increasing NaOH concentration, reaching 93.26% at 3 mol/L NaOH concentration and further increasing to 98.21% when the NaOH concentration increases to 4 mol/L. This occurs because as the alkalinity increases, the probability of collision between tin and alkali increases and the dissolution efficiency of tin increases. The results of the thermodynamic study, as shown in Figure 1f, also demonstrate that the potential for the oxidation of tin to sodium stannate decreases with increasing pH, facilitating the dissolution of tin and its conversion to sodium stannate. Upon further increasing the alkali concentration to 5 or 6 mol/L, the tin dissolution efficiency remains relatively constant at approximately 99.88%. However, an increase in the NaOH concentration leads to a significant increase in cost. Consequently, a sodium hydroxide concentration of 4 mol/L is selected as the optimal condition.

#### 2.1.5. Effect of Ultrasonic Time

The influence of ultrasonic time on the tin dissolution efficiency during the ultrasonic tin dissolution process was examined under the following conditions: 4 mol/L NaOH concentration, two times the theoretical amount of H_2_O_2_ dosage, room temperature (20 °C), and 240 W ultrasonic power. The dissolution efficiency of Sn with different ultrasonic times is shown in Figure 1e.

Extending the ultrasonic time is beneficial for the decomposition of hydrogen peroxide into hydroxyl radicals, thereby enhancing the efficiency of tin dissolution. Hu et al. [10] demonstrated that the generation of hydroxyl radicals in ultrasonically treated aqueous solutions, as measured by chemiluminescence, was positively correlated with the duration of sonication. The quantity of hydroxyl radicals increased with the extension of ultrasonic time [10]. Furthermore, another study also reported that in aqueous solutions, an extended sonication duration facilitates the production of hydroxyl radicals [14]. In our work, the tin dissolution efficiency reached 99.3% after ultrasonic treatment for 60 min, and upon further extending the treatment to 70 min, the tin dissolution efficiency is not much different, which is 99.7%. Consequently, an ultrasonic treatment duration of 60 min is identified as the optimal reaction condition.

In summary, the optimal process parameters for the ultrasonic room-temperature dissolution of tin are as follows: room temperature (20 °C), ultrasonic power of 240 W, hydrogen peroxide addition of twice the theoretical amount, a sodium hydroxide concentration of 4 mol/L, and an ultrasonic time of 60 min. Under these optimal conditions, the tin dissolution efficiency reached up to 99.3%.

#### 2.1.6. Comparison of Tin Dissolution at Maximum Efficiency (>99%) Under Ultrasonic and Conventional Conditions

Based on the above optimal conditions, at room temperature (20 °C), hydrogen peroxide addition of twice the theoretical amount, and a sodium hydroxide concentration of 4 mol/L, the tin dissolution efficiencies under ultrasonic and conventional conditions were compared. The results are listed in Table 1.

Without ultrasound, the tin dissolution efficiency is only 71.3% after 60 min of reaction at room temperature, which is 28% lower than that of the ultrasonic dissolution under the same conditions. In addition, by optimizing the conditions for conventional tin dissolution, the tin dissolution efficiency of conventional conditions is found to reach 99.1% when the temperature is 50 °C, the concentration of sodium hydroxide is 6 mol/L, the concentration of hydrogen peroxide is 30%, and the amount of hydrogen peroxide added is 2.5 times the theoretical value. This tin dissolution effect is basically the same as the 99.3% effect under the ultrasonic optimization conditions introduced above, which shows that the introduction of ultrasound can decrease the reaction temperature by 30 °C, reduce the consumption of sodium hydroxide by 33.3%, and save the consumption of hydrogen peroxide by 15%, which effectively reduces the energy consumption and the consumption of reactants in the tin oxidation process.

### 2.2. Mechanism of Ultrasound-Enhanced Tin Dissolution at Room Temperature

#### 2.2.1. Increasing the Generation of Free Radicals with Ultrasound

**Figure 3:**
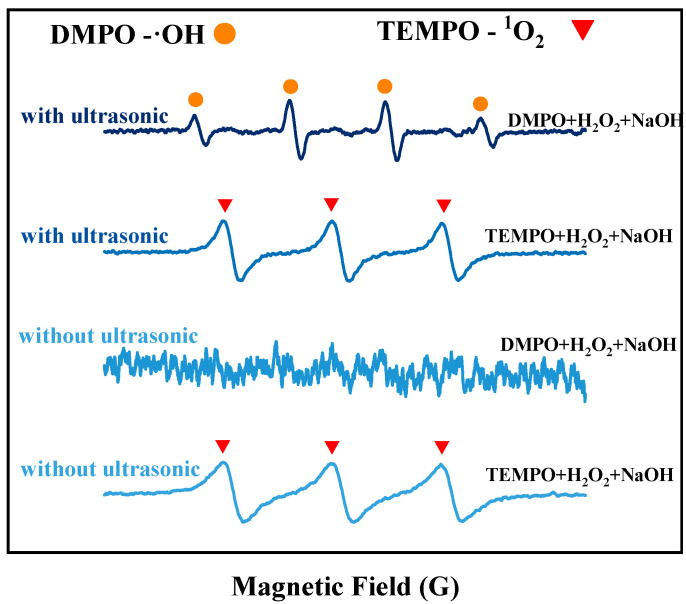
EPR detection results under ultrasonic and conventional conditions.

The EPR detection method was used to assess the generation of free radicals from H_2_O_2_ in an alkaline solution at room temperature, in the absence of tin sheets, under ultrasonic and non-ultrasonic conditions. The results are shown in Figure 3. Without ultrasound, no obvious signal peaks for hydroxyl radicals (OH) are observed in the solution, and only the signal peak for singlet oxygen (^1^O_2_) is detected. However, under the action of ultrasound, both ·OH and ^1^O_2_ signal peaks are detected, and the peak intensity of the ·OH signal peak is significantly increased. The reason is that without ultrasound, few ·OH are generated in the solution, and these ·OH are quickly consumed by hydroxide ions (OH^−^), leading to the formation of superoxide radicals (O^2−^), as shown in Equation (5) [15]. These ·O^2−^ radicals rapidly react with the hydrogen peroxide present in the solution to form ^1^O_2_, as shown in Equation (6). Research by Almeida et al. [16] indicated that alkaline conditions were conducive to the generation of singlet ^1^O_2_ from H_2_O_2_. After the addition of ultrasound, the ·OH and ^1^O_2_ signal peaks are detected, with a notably enhanced signal peak for ·OH. This is because more ·OH radicals are produced by the decomposition of H_2_O_2_ under the action of ultrasound, which cannot be completely consumed by OH^−^ in the solution, leading to the detection of a distinct ·OH signal peak. The introduction of ultrasound enhances the formation of ·OH at room temperature, and the redox potential of ·OH (2.8 V) [17] is higher than that of H_2_O_2_ (1.78 V) [18] and ^1^O_2_ (2.2 V) [19]. As shown in Figure 3, the peak intensity of the ·OH signal peak increases with increasing ultrasonic power. To elucidate the contribution of ·OH to the oxidation process, tert-butyl alcohol (TBA) was employed as a ·OH scavenger [15] in our study. Under optimal conditions for ultrasonic tin dissolution, the tin dissolution efficiency reached 99.3% in the absence of TBA. However, when TBA was introduced to scavenge ·OH radicals, the tin dissolution efficiency significantly decreased to 61.52%, confirming the significant role of hydroxyl radicals in the ultrasonic tin dissolution process. This results in an increased oxidizing capacity of H_2_O_2_, thereby enhancing the tin dissolution efficiency [19].

H_2_O_2_ ↔ 2·OH (4)

2·OH +2OH^−^ → ·O_2_^−^ + 2H_2_O (5)

·O_2_^−^ +·OH → ^1^O_2_ + OH^−^ (6)

#### 2.2.2. Increasing the Surface Roughness of Tin Sheets with Ultrasound

**Figure 4:**
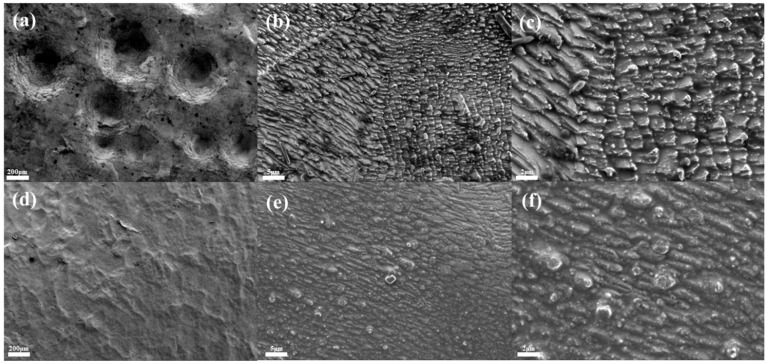
SEM images of tin sheet surfaces after ultrasonic treatment (**a**–**c**) and after conventional treatment (**d**–**f**).

Figure 4a–c show the SEM images of tin sheets under ultrasonic action. Ultrasonic treatment results in the formation of numerous concave structures on the tin sheet surface, which significantly increase the surface roughness and even cause perforation in some areas. The rough surface topography increases the contact area between the solid and the liquid, thereby promoting the further dissolution of the tin sheet. In comparison, the surface of the tin sheet treated under conventional conditions, as shown in Figure 4d–f, is relatively smooth, without significant concave structures, and exhibits a lower surface roughness, which is less favorable for solid–liquid contact and enhances reaction efficiency.

**Figure 5:**
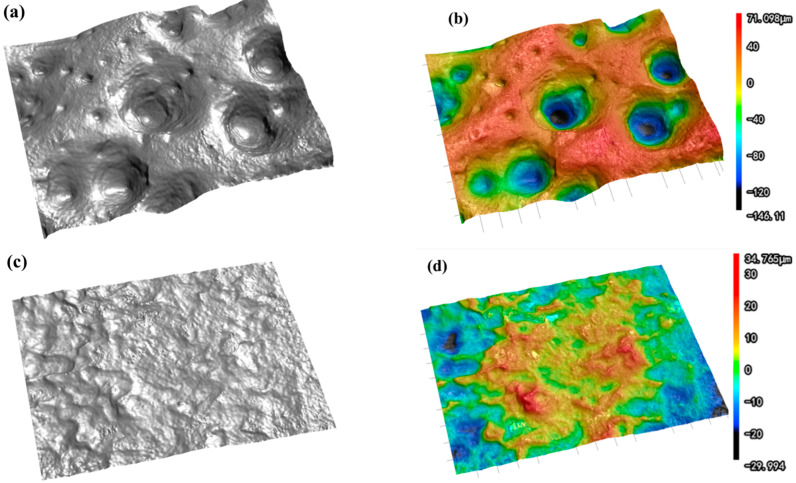
Optical profilometer scans of the tin sheets after ultrasonic treatment (**a**,**b**) and after conventional treatment (**c**,**d**).

As shown in Figure 5, the optical profilometer test results reveal that a large number of pores appear on the surface of the tin sheet after ultrasonic treatment, which is consistent with the SEM test results. The maximum height difference of the surface is an important indicator for measuring surface roughness, which represents the distance between the peak line and the valley line of the surface profile. The maximum height difference on the tin sheet surface significantly increased from 41.134 μm after conventional treatment to 303.074 μm after ultrasonic treatment. The average surface roughness is the average value of the absolute value of the distance from each point on the surface contour line to the reference line within the specified sampling length. The larger the average roughness value, the rougher the surface, with more tiny peaks and deeper grooves. The average surface roughness of the tin sheet is 6.875 μm after conventional treatment and 34.135 μm after ultrasonic treatment. After ultrasonication, the surface roughness of the tin sheet is significantly increased, which contributes to increasing the solid–liquid contact area and accelerating the reaction efficiency.

**Figure 6:**
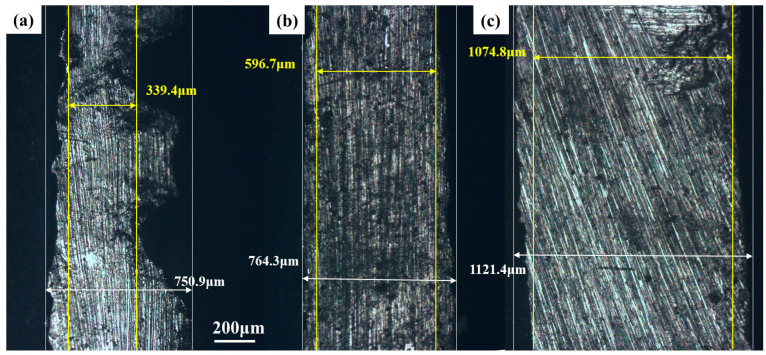
Thicknesses of the tin sheets after ultrasonic treatment (**a**); after conventional treatment (**b**); raw material tin sheet (**c**).

The changes in the thickness of tin sheets after ultrasonic and conventional treatments were analyzed using a metallographic microscope, and the results are shown in Figure 6. After 30 min of treatment, the thickness of the tin sheet after conventional treatment is 596 μm at the minimum and 764.3 μm at the maximum, whereas the thickness of the tin sheets treated with ultrasound is 339.4 μm at the minimum and 750.9 μm at the maximum. At the same time, the impact of ultrasonic treatment is obvious, making the tin sheets significantly thinner and accelerating the dissolution process faster.

#### 2.2.3. Elimination of Passivation Layer on the Surface of Tin Sheets with Ultrasound

**Figure 7:**
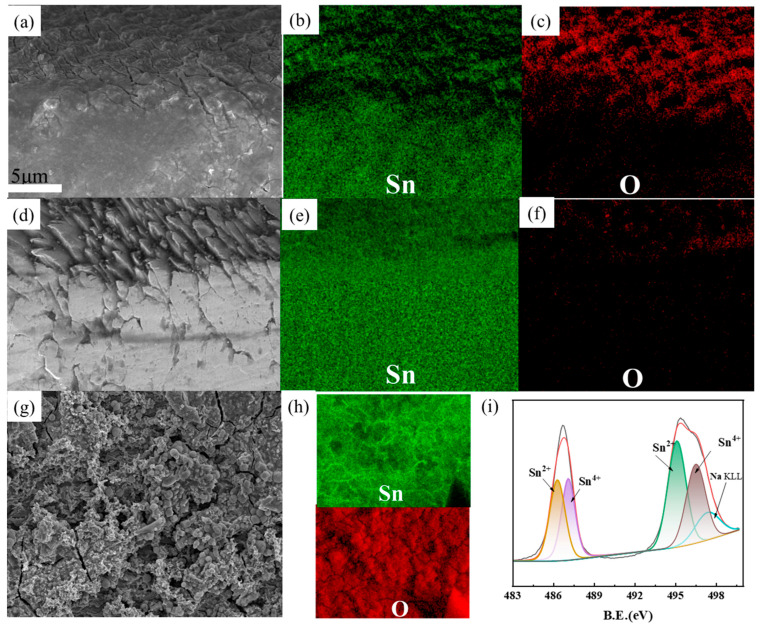
SEM images of the tin sheet passivation interface at conventional treatment (**a**); distribution of tin elements at the passivation interface under conventional conditions (**b**); distribution of oxygen elements at the passivation interface under conventional conditions (**c**); SEM images of the tin sheet passivation interface at ultrasonic treatment (**d**); distribution of tin elements at the passivation interface under ultrasonic conditions (**e**); distribution of oxygen elements at the passivation interface under ultrasonic conditions (**f**); SEM images of the tin sheet surface at conventional treatment (**g**); distribution of tin and oxygen elements at the passivation interface under ultrasonic conditions (**h**); results of the XPS analysis of the tin sheet surface at conventional treatment (**i**).

As shown in Figure 7a, an extremely thin passivation layer forms on the surface of the tin sheet. This layer impedes the contact between the tin sheet and the solution, thereby directly limiting the rate of tin oxidation. Elemental distribution comparisons (Figure 7b,c) reveal a substantial presence of oxygen and tin on the tin sheet surface, indicating that the passivation layer is primarily composed of incomplete tin oxides. In contrast, the surface of the tin sheet treated with ultrasound exhibits a uniform distribution of tin elements, with no distinct mixture of tin and oxygen elements uniformly distributed (Figure 7d–f). This suggests that the passivation layer is disrupted by the ultrasonic action, facilitating contact between the tin sheet and the oxidizing solution and thereby enhancing the oxidation process.

As shown in Figure 7g, the passivation layer forms on the surface of the tin flake under conventional treatment conditions. As shown in Figure 7h, both tin and oxygen elements are uniformly distributed across the sample surface. XPS analysis of the surface of the passivation layer indicates that the tin element primarily consists of Sn^2+^ and Sn^4+^ in Figure 7i. The characteristic peaks at 486.3 eV and 494.7 eV are mainly attributed to Sn^2+^, and the peaks at 495.1 eV and 487.1 eV are mainly attributed to Sn^4+^ [20]. The oxides of divalent and tetravalent tin constitute the main components of the passivation layer. Additionally, a small amount of sodium ions from the solution (496.5 eV) are also interspersed on the surface of the passivation layer.

**Figure 8:**
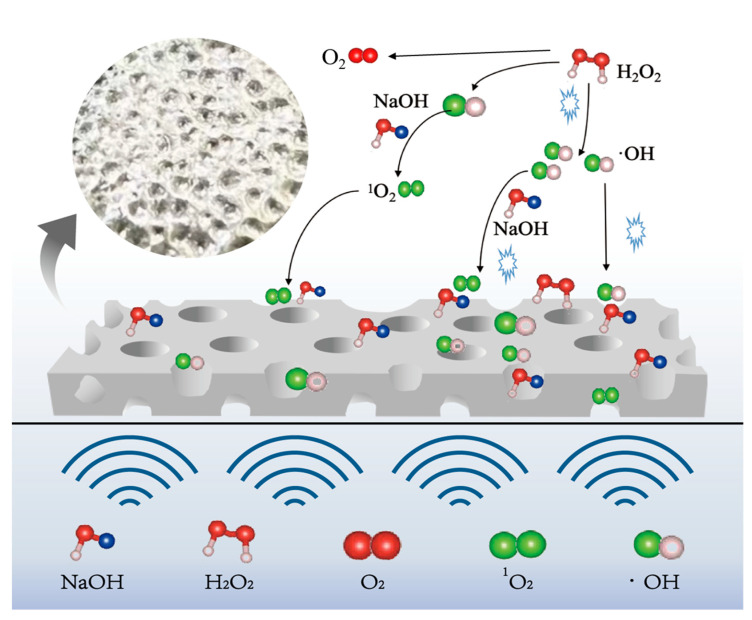
Mechanism diagram of ultrasonic enhancement.

The mechanism of ultrasonic enhancement on the tin dissolution process at room temperature is depicted in Figure 8. Ultrasound can also promote the generation of hydrogen peroxide into more oxidizing ·OH at room temperature conditions, providing a higher oxidation potential for the system, promoting the dissolution of tin, and increasing the utilization rate of H_2_O_2_. The micro-jets generated by ultrasound play a scouring role on the surface of the tin sheet and promote the etching of the tin sheet surface by NaOH and H_2_O_2_, enhancing surface roughness and effectively increasing the solid–liquid contact area. And ultrasound removes the passivation layer on the surface of the tin sheet, which is beneficial for the contact between the tin sheet and hydrogen peroxide and alkali. Consequently, ultrasound can increase the tin dissolution efficiency to 99.3% at room temperature, which is 28% higher than that of the conventional tin dissolution under the same conditions. The introduction of ultrasound offers advantages in lowering the reaction temperature and reducing the consumption of sodium hydroxide and hydrogen peroxide. In this technology, the reactants are nearly fully converted into sodium stannate, and H_2_O_2_ decomposes only into oxygen, which is environmentally benign. Undissolved tin is non-toxic and can be recycled in subsequent batches. In summary, this technology has no harmful environmental effects.

The enhancement effects of ultrasound in oxidation dissolution processes for other metals have been investigated by our research team. For example, in the treatment of copper anode mud, the copper dissolution efficiency was increased from 66.64% to 98.11% through ultrasound activation when ammonium persulfate was employed as the oxidant [21]. For copper–cadmium slag [22], ultrasonic-enhanced ozone oxidation improved copper dissolution from 45.7% to 99.3%, with cadmium dissolution reaching over 99.4%. In the case of zinc oxide dust processing [15], ultrasound increased Zn and Ge leaching rates by 7.86% and 5.65%, respectively. These results demonstrate the broad applicability of ultrasound-assisted oxidation dissolution across multiple metal systems. Ultrasonic intensification technology at industrial scale significantly enhances metal dissolution efficiency while reducing reagent consumption and operational costs [23]. However, to replicate the superior performance achieved in laboratory-scale tests, industrial applications require improved power density and energy utilization efficiency through measures such as enhanced equipment corrosion resistance and optimized reactor layout design for ultrasonic devices.

## 3. Materials and Methods

### 3.1. Materials

**Figure 9:**
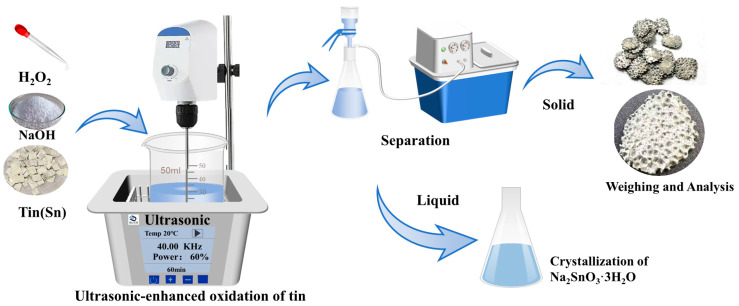
Schematic diagram of the experimental equipment.

The NaOH used in this experiment was produced by Tianjin Zhiyuan Chemical Reagent Co., Ltd. (Tianjin, China). The tert-butanol (TBA) was produced by Tianjin Zhiyuan Chemical Reagent Co., Ltd. High-purity tin sheets were produced by Tengfeng Metal Materials Co., Ltd. (Xingtai, China), with a thickness of 1 mm and a purity of 99.99%. The 30% H_2_O_2_ used in this experiment was obtained from Tianjin Zhiyuan Chemical Reagent Co., Ltd. (Tianjin, China) The distilled water utilized in the experiment was produced in-house in the laboratory. The ultrasonic equipment used was homemade by the Key Laboratory of Unconventional Metallurgy. The ultrasonic frequency was 40 kHz, the ultrasonic waves were emitted from the bottom, and the ultrasonic power was regulated by adjusting the output power percentage with a maximum output power of 300 W. The experimental setup is shown in Figure 9.

### 3.2. Experimental Principles and Procedure

The experimental principle involves the dissolution of tin sheets in a NaOH solution with H_2_O_2_ as the oxidant, resulting in the formation of a Na_2_SnO_3_ solution. The chemical reaction is depicted in Equation (1).

Commercial tin sheets were crushed into small pieces measuring 0.6 cm × 0.6 cm. NaOH and H_2_O_2_ solutions were prepared to the required concentrations for the experiment. A total of 50 mL of the prepared NaOH solution was poured into a beaker and then placed in an ultrasonic generator. A stirring paddle was added to the beaker, and the stirring was initiated. Subsequently, 4.72 g of the crushed tin pieces were added to the solution. After the ultrasound was turned on, a hydrogen peroxide solution was gradually added to the solution. To ensure the utilization efficiency of the solution, the hydrogen peroxide solution was slowly introduced at a rate of 1 mL per minute. After the reaction was complete, any unreacted high-purity tin was separated from the solution, rinsed with deionized water and anhydrous ethanol, and then quickly dried. The tin dissolution efficiency was calculated based on the change in mass of the tin sheets, as shown in Equation (2).

(7) $$p=\frac{m_{0}-m_{1}}{m_{0}}\times 100\%$$

where, p represents the dissolution efficiency, $m_{0}$ denotes the mass of the tin sheets before dissolution, and $m_{1}$ (g) denotes the mass of the tin sheets after dissolution. To minimize experimental errors, each experiment was conducted three times. Owing to the complexity of the reaction system, which involves multiple solution variables, the stirring rate was fixed at 300 rpm, and the hydrogen peroxide concentration was 15% according to actual production conditions. Using tert-butanol (TAB) as a scavenger of ·OH, the effect of ·OH generated by ultrasound on tin oxidation under optimized experimental conditions was investigated.

### 3.3. Characterization Equipment

SEM-EDS (ZEISS Sigma 300, Carl Zeiss AG, Oberkochen, Germany) was used to analyze the surface morphology of the tin sheet. An optical profilometer (Mahr MarSurf LD130, Mahr GmbH, Göttingen, Germany) was utilized to investigate the sample surface and to acquire roughness data. Zeiss ordinary inverted fluorescence microscope (Zeiss, Axio Vert. A1, Carl Zeiss AG, Oberkochen, Germany) was employed to analyze variations in sample thickness. An electron paramagnetic resonance spectrometer (EPR JES-FA200, JEOL, Tokyo, Japan) was used to analyze free radicals in the solution, employing the DMPO spin-trapping EPR technique with 5,5-dimethyl-1-pyrrolidine N-oxide (DMPO) and 2,2,6,6-tetramethylpiperidine (TEMP) as spin traps, thereby further validating the generation of free radicals. ·OH and ·O_2_^−^ can react with DMPO to form DMPO-·OH and DMPO-·O_2_^−^ adducts, and ^1^O_2_ can react with TEMPO to form TEMPO-^1^O_2_ adducts [15].

## 4. Conclusions

In this work, the effect of ultrasound on dissolving tin at room temperature was investigated, and the mechanism of ultrasound strengthening was revealed. The application of ultrasound enabled efficient dissolution of tin sheets at room temperature without heating. Under room temperature (20 °C), 240 W ultrasonic power, two times the theoretical amount of hydrogen peroxide addition, 4 mol/L sodium hydroxide concentration, and a 60 min reaction time, the dissolution efficiency of tin sheets reached 99.3%, which was 28% higher than that of conventional tin dissolution efficiency under the same conditions. Compared with conventional conditions that achieve the same dissolution effect, the introduction of ultrasonic technology reduced the reaction temperature by 30 °C, sodium hydroxide consumption by 33.3%, and hydrogen peroxide consumption by 15%, greatly reducing the energy consumption and chemical usage in the tin oxidation process.

The mechanism of ultrasound enhancement is primarily manifested in three aspects: (1) Ultrasonication promoted the generation of more oxidizing ·OH with stronger oxidizing properties from H_2_O_2_ at room temperature, providing a higher oxidation potential for the reaction system and facilitating oxidative dissolution of tin. (2) The surface roughness of the tin sheets significantly increased after ultrasonic treatment, from 6.875 μm in conventional treatment to 34.135 μm with ultrasound, and the maximum height difference on the tin sheet surface increased from 41.134 μm in conventional treatment to 303.074 μm in ultrasonic treatment, effectively expanding the solid–liquid contact area and enhancing the dissolution efficiency of the tin sheets. (3) Ultrasound destroyed the tin oxide passivation layer formed on the surface of the tin sheet, thereby enhancing the contact between the tin and the NaOH/H_2_O_2_ solution, which in turn intensified the oxidative dissolution reaction of the tin sheet. This study developed a new process of ultrasound-enhanced tin dissolution at room temperature, which solved the problems of low efficiency, high operating temperatures, and low utilization efficiency of hydrogen peroxide in conventional tin dissolution technology.

## Figures and Tables

**Table 1:** Tin dissolution at maximum efficiency (>99%) under ultrasonic and conventional conditions.

Conditions of Dissolution	Conventional	Ultrasonic
temperature	50 °C	20 °C
NaOH concentration	6 mol/L	4 mol/L
H_2_O_2_ concentration	30%	15%
H_2_O_2_ addition amount/theoretical amount	2.5 times	2.0 times

## Data Availability

Data is contained within the article.
